# Targeting MDK Abrogates IFN-γ-Elicited Metastasis inCancers of Various Origins

**DOI:** 10.3389/fonc.2022.885656

**Published:** 2022-06-07

**Authors:** Luyu Zheng, Qun Liu, Ruijun Li, Shibin Chen, Jingyu Tan, Lina Li, Xichen Dong, Changzhi Huang, Tao Wen, Jian Liu

**Affiliations:** ^1^ Medical Research Center, Beijing Chao-Yang Hospital, Capital Medical University, Beijing, China; ^2^ Department of Obstetrics and Gynaecology, Beijing Anzhen Hospital, Capital Medical University, Beijing, China; ^3^ State Key Laboratory of Molecular Oncology, Beijing Key Laboratory for Carcinogenesis and Cancer Prevention, Department of Etiology and Carcinogenesis, National Cancer Center/Cancer Hospital, Chinese Academy of Medical Sciences and Peking Union Medical College, Beijing, China; ^4^ Department of Oncology, Beijing Chao-Yang Hospital Capital Medical University, Beijing, China

**Keywords:** cancer, IFN-γ, MDK, epithelial-to-mesenchymal transition, metastasis

## Abstract

IFN-γ is a pleiotropic cytokine with immunomodulatory and tumoricidal functions. It has been used as an anti-tumor agent in adjuvant therapies for various cancers. Paradoxically, recent advances have also demonstrated pro-tumorigenic effects of IFN-γ, especially in promoting cancer metastasis, with the mechanism remains unclear. This will undoubtedly hinder the application of IFN-γ in cancer treatment. Here, we verified that IFN-γ treatment led to activation of the epithelial-to-mesenchymal transition (EMT) programme and metastasis in cell lines of various cancers, including the kidney cancer cell line Caki-1, the lung cancer cell line A549, the cervical carcinoma cell line CaSki, the breast cancer cell line BT549 and the colon cancer cell line HCT116. We further disclosed that midkine (MDK), an emerging oncoprotein and EMT inducer, is a common responsive target of IFN-γ in these cell lines. Mechanistically, IFN-γ upregulated MDK *via* STAT1, a principle downstream effector in the IFN-γ signalling. MDK is elevated in the majority of cancer types in the TCGA database, and its overexpression drove EMT activation and cancer metastasis in all examined cell lines. Targeting MDK using a specific MDK inhibitor (iMDK) broadly reversed IFN-γ-activated EMT, and subsequently abrogated IFN-γ-triggered metastasis. Collectively, our data uncover a MDK-dependent EMT inducing mechanism underlying IFN-γ-driven metastasis across cancers which could be attenuated by pharmacological inhibition of MDK. Based on these findings, we propose that MDK may be used as a potential therapeutic target to eliminate IFN-γ-elicited pro-metastatic adverse effect, and that combined MDK utilization may expand the application of IFN-γ in cancer and improve the clinical benefits from IFN-γ-based therapies.

## Introduction

Interferons (IFNs) constitute a family of cytokines that have antiviral, antiproliferative and immunomodulatory properties (1). There are three main classes of cytokines in the IFN family: IFN-I (IFN-α, β, ϵ, κ, and ω), IFN-II (IFN-γ), and IFN-III (IFN-λ1, λ2, λ3, and λ4) (2). These cytokines play pivotal roles in host defense against viral and bacterial infections, as well as immunosurveillance for malignant cells (3).

IFN-γ, encoded by the gene *IFNG*, is the only member of IFN-II. It is a pleiotropic cytokine with a long history of clinical trials in cancer treatment (4, 5). Since the first clinical trial conducted in 1985 (6), the therapeutic application of IFN-γ has been tested in a variety of malignancies, including melanoma, leukemia, ovarian cancer, renal cell carcinoma, hepatocellular carcinoma, lung cancer, breast cancer, bladder cancer and colorectal cancer (7). Clinical benefits derived from IFN-γ-based therapies have been reported in several cancers (8–10), highlighting the therapeutic value of IFN-γ in combating cancers.

IFN-γ exerts anti-tumor effects by boosting antitumor immunity and by direct effects on cancer cells (1, 11). IFN-γ enhances the activity of cytotoxic CD8 T cells, NK cells, Th1 cells, dendritic cells and macrophages; stimulates the expression of the major histocompatibility complex (MHC) class I and II molecules in tumor cells and APCs; promotes differentiation of macrophages towards a pro-inflammatory (M1-like) phenotype; and bridges the innate and adaptive immune responses (3, 12, 13). IFN-γ also exerts direct cytotoxic effects on neoplastic cells through anti-proliferative, anti-angiogenic and pro-apoptotic mechanisms (7, 14, 15).

Despite these anti-tumor activities, IFN-γ has been paradoxically reported to increase the risk of tumor metastasis (16–22). This pro-tumorigenic activity has been reported in colon adenocarcinoma (16), non-small cell lung cancer (20), prostate cancer (17), renal cancer (18), triple-negative breast cancer (21), and melanoma (19, 22) *via* multiple mechanisms. However, the mechanisms underlying IFN-γ-induced metastasis remain unclear, and whether there is a shared mechanism mediating IFN-γ-induced metastasis in cancers of different origins is still unknown.

Here, we reveal, for the first time, that the EMT inducer MDK, is a common responsive target of IFN-γ in all examined five cancer cell lines, and that MDK confers the pro-metastatic function of IFN-γ in these cell lines by activating the EMT programme; while pharmacologically targeting MDK using a specific inhibitor globally attenuated IFN-γ treatment-induced EMT and metastasis in all examined cancers. We thus propose that blocking the pro-tumorigenic activities of IFN-γ using MDK inhibitors may help to improve the clinical benefits from IFN-γ-based therapies.

## Materials and Methods

### Cell Lines and Culture

The human renal cancer cell line Caki-1, human lung cancer cell line A549, human colorectal adenocarcinoma cell line HCT116, cervical cancer cell line CaSki, human breast cancer cell line BT549 and human embryonic kidney cell line HEK293T were acquired from National Collection of Authenticated Cell Cultures (Beijing, China). Caki-1, A549 and HCT116 cells were maintained in McCoy’s 5A medium modified (KeyGEN BioTECH, Jiangsu, China). CaSki and BT549 cells were cultured in RPMI-1640 medium (SIGMA, Vienna, Austria). HEK293T were maintained in DMEM medium (Gibco, California, USA). All mediums contained 10% fetal bovine serum (FBS, Ausbian, Australia) and 1% penicillin–streptomycin mixture (Solarbio, Beijing, China). All cell lines were incubated at 37°C with 5% CO_2_ in a humidified incubator.

### Antibodies and Reagents

MDK antibody (1:1000 dilution, 11009-1-AP) was purchased from Proteintech Group Inc (Rosemont, IL, USA). E-cadherin antibody (1:1000 dilution, 3195T), ZO1 antibody (1:1000 dilution, 8193T), Vimentin antibody (1:1500 dilution, 5741T) and Snail antibody (1:500 dilution, 3879T) were from Cell Signaling Technology (Danvers, Massachusetts, USA). STAT1 antibody (1:5000 dilution, ab109320) and phospho-STAT1 (phosphor Y701) antibody (1:1000 dilution, ab109457) were purchased from Abcam (Cambridge, UK). β-actin antibody (1:1000 dilution, AF5001) was from Beyotime Biotechnology (Shanghai, China). Recombinant human IFN-γ was acquired from PeproTech (Rocky Hill, NJ, USA) and used at a concentration of 50 ng/ml for 48h. The MDK inhibitor iMDK was bought from Merck Millipore (Darmstadt, Germany) and diluted to a final concentration of 100nM in growth mediums. The STAT1 inhibitor Fludarabine was purchased from Med Chem Express (New Jersey, USA) and used at a concentration of 5 μM for 48h.

### RNA Isolation and Quantitative Real-Time PCR (qRT-PCR)

Total RNA was extracted using TRIeasy reagent (Yeasen Biotechnology, Shanghai, China) according to the manufacturer’s instruction. The concentration and purity of RNA were determined by Nanodrop 1000. The ratio of OD 260/OD 280 falling between 1.8 and 2.0 indicates acceptable values. Reverse transcription was achieved with a total of 1 μg RNA using the cDNA Synthesis SuperMix (Yeasen Biotechnology, Shanghai, China). The primers (5′-3′) synthesized by Rui Biotech (Beijing, China) were listed in **Table S1**. QRT-PCR was performed using qPCR SYBR Green Master Mix (Yeasen Biotechnology, Shanghai, China). Each sample was run in 3 duplicate wells, and the relative RNA expression levels between different samples were analyzed by 2 ^–ΔΔCt^ method, with β-actin as an internal control.

### Lentivirus Production and MDK Overexpression

The human MDK overexpression lentiviral vector (YOE-LV004-hMDK) with neomycin resistance and the corresponding control vector (YOE-LV004-Ctrl) were purchased form UBIGENE (Guangzhou, China). Lentivirus was produced in HEK293T cells by co-transfecting the lentiviral vector with the psPAX2 and pMD2G packaging vectors using Lipofectamine 3000 (Invitrogen, Carlsbad, CA, USA). Caki-1, A549, HCT116, CaSki and BT549 cells were infected with the packaged lentiviruses with polybrene, and then selected with G418 (Solarbio, Beijing, China) at a concentration of 400 μg/ml. The empty plasmid (YOE-LV004-Ctrl) was used as a control.

### Transwell Assay

Transwell assays were used to assess the invasion and migration abilities of cancer cells. Briefly, 1×10^5^ cells in serum-free medium were seeded into the upper chamber of transwell plates (Corning, NY, USA), while the lower chamber was added with 10% FBS medium. For invasion assay, the transwell chambers were pre-coated with Matrigel (BD Biosciences, Palo Alto, CA). Approximately 16-24 h after seeding, the cells remaining on the upper chamber were carefully wiped off with cotton swabs, while the migrated cells were fixed in 4% paraformaldehyde for 20 minutes and then stained in 0.2% crystal violet solution for 20-25 minutes. After cleaning the chamber with wash buffer for three times, the migrated cells were counted under an inverted bright-field microscope.

### Western Blotting Assay

Cell lysates were prepared in RIPA lysis buffer (Beyotime Biotechnology, Shanghai, China) supplemented with 1x protease inhibitor cocktail (Beyotime Biotechnology) and 1x phenylmethylsulfonyl fluoride (PMSF, APPLYGEN, Beijing, China). Protein concentrations were measured with a BCA assay kit (KeyGEN BioTECH, Jiangsu, China). The protein samples (50 μg) were boiled at 100°C for 10 minutes, separated by 10-12% SDS-PAGE, and then transferred onto PVDF membranes (Merck Millipore, MA, USA) *via* semi-dry transfer unit. After blocked in 5% skim milk at room temperature for 1h, the membranes were incubated overnight at 4°C with primary antibodies, and then incubated with anti-rabbit or mouse IgG-HRP secondary antibody for 2h at room temperature. Membranes were visualized using Baygene Chemilmaging system (Baygene Biotech, Beijing, China) with Super ECL Detection Reagent (Yeasen Biotechnology, Shanghai, China).

### Statistical Analysis

All statistical analysis was performed in GraphPad Prism 8.0 (GraphPad Software, La Jolla, CA, USA). Unpaired and multiple T-test were used to assess the differences between control and experimental groups. P value < 0.05 was considered statistically significant. The results were presented as mean ± standarddeviation (SD). *P<0.05, **P<0.01, ***P<0.001, ****P<0.0001.

## Results

### IFN-γ Treatment Enhances EMT and Metastasis in Various Tumors

**Figure 1:**
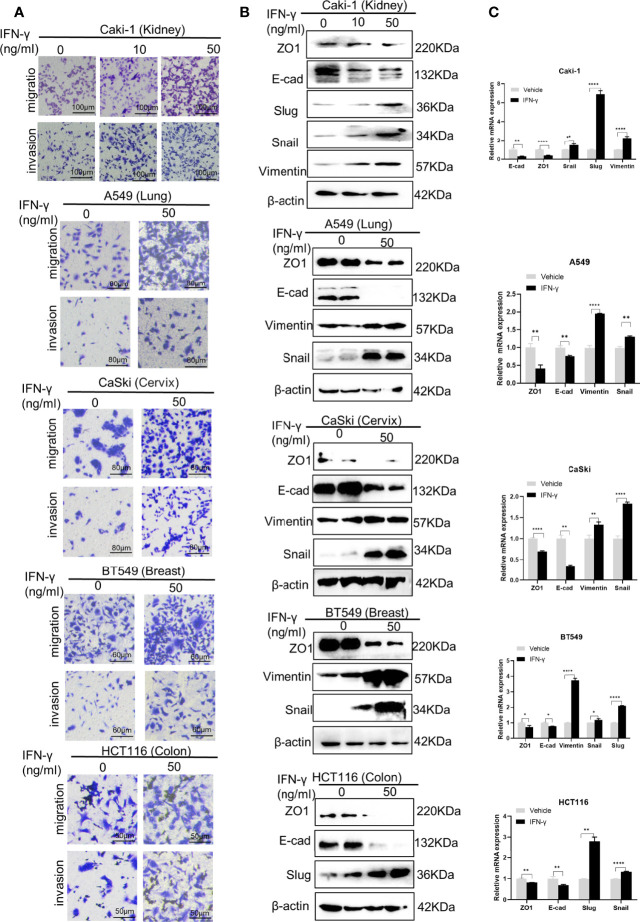
IFN-γ treatment enhances epithelial-mesenchymal transition (EMT) and metastasis in various tumors. **(A)** Transwell assays to detect the migration and invasion abilities of different cancer cell lines without and with IFN-γ treatment (50 ng/ml, 48 h). **(B)** Western blotting assays to examine the effect of IFN-γ treatment (50 ng/ml, 48 h) on expression of EMT markers at the protein level. **(C)** RT-qPCR assays to detect the changes in expression of EMT markers at the mRNA level after IFN-γ treatment (50 ng/ml, 48 h). Data are shown as the mean ± standard deviation (SD). *P < 0.05, **P < 0.01, ****P < 0.0001.

Interferon-gamma (IFN-γ) is a pleiotropic cytokine with antiproliferative, pro-apoptotic and immunomodulatory functions. IFN-γ has been used to treat a variety of malignancies in pre-clinical and clinical trials; however, limited benefits have been achieved, presumably due to its pro-tumor adverse effects, including eliciting metastasis (23). Prior to deciphering the mechanism underlying IFN-γ-induced cancer metastasis, we first verified the pro-metastatic effect of IFN-γ in five cell lines of different human cancers (**Figure 1A**): the kidney cancer cell line Caki-1, the lung cancer cell line A549, the cervical carcinoma cell line CaSki, the breast cancer cell line BT549, and the colon cancer cell line HCT116. Transwell assays demonstrated that IFN-γ exposure obviously increased the migration and invasion abilities of all the five cancer cell lines (**Figure 1A**). This is in line with the previous findings in colon cancer (16), prostate cancer (17), non-small cell lung cancer (20), and melanoma (19), suggesting that IFN-γ treatment could globally enhance metastasis of cancers.

EMT is a common mechanism driving metastasis of cancers (24). We then examined the role of IFN-γ in the EMT programme. Western blotting results showed that IFN-γ treatment led to decreased expression of epithelial markers, including ZO-1 and E-cadherin, and concomitant upregulation of mesenchymal markers, such as Vimentin, Snail and Slug in the above cell lines at the protein level (**Figure 1B**). The alterations in expression of EMT markers were further verified at the mRNA level (**Figure 1C**). These data indicate that IFN-γ may promote metastasis by activating the EMT programme in cancers.

### IFN-γ Exposure Promotes MDK Expression in Cancers

**Figure 2:**
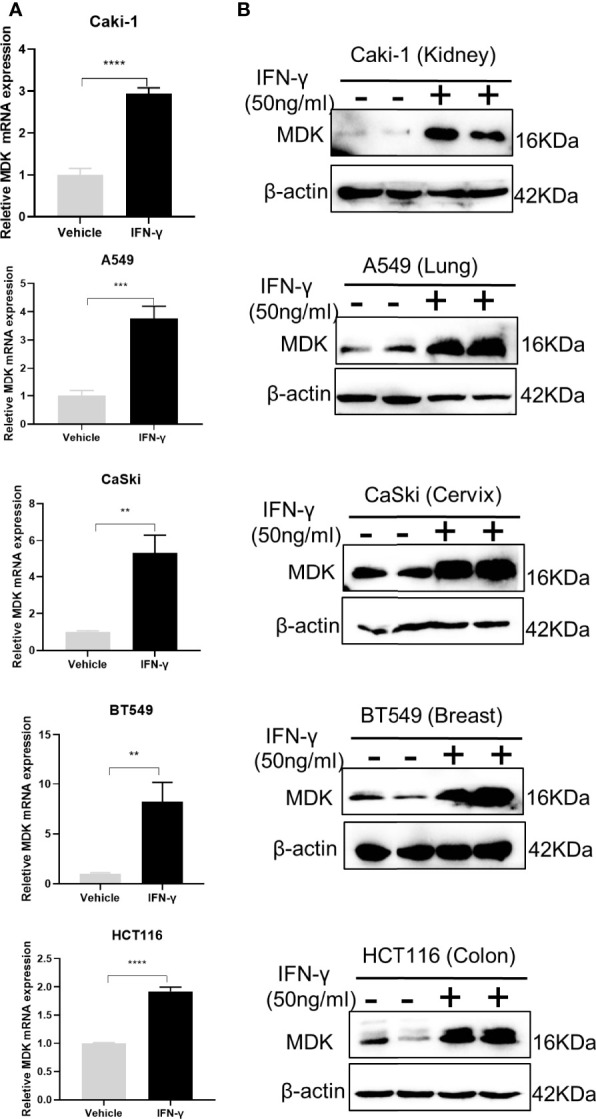
IFN-γ treatment promotes MDK expression in different cancers. **(A)** RT-qPCR assays to detect the expression of MDK in five cancer cell lines without and with IFN-γ treatment (50 ng/ml, 48 h). **(B)** Western blotting assay to determine the influence of IFN-γ treatment (50 ng/ml, 48 h) on MDK protein levels in five cancer cell lines. Data are shown as the mean ± standard deviation (SD). **P < 0.01, ***P < 0.001, ****P < 0.0001.

MDK is a heparin-binding growth factor well-documented to promote EMT and cancer metastasis (25). Though there is no evidence suggesting the regulatory relationship between IFN-γ and MDK in the cancerous context, we found a previous report indicating that the MDK expression in lymphocytes is upregulated in response to IFN-γ treatment (26). This gave rise to the speculation that whether IFN-γ treatment in cancers would result in MDK activation, thereby triggering EMT and metastasis. To verify this hypothesis, we treated different cancer cell lines with IFN-γ (50 ng/ml) and then examined the alterations of MDK expression in the mRNA and protein levels. Real-time qPCR assays demonstrated a dramatic upregulation of MDK mRNA by IFN-γ in all examined cancer cell lines (**Figure 2A**), including the kidney cancer Caki-1, the lung cancer A549, the cervical carcinoma CaSki, the breast cancer BT549, and the colon cancer HCT116, which was further validated by Western blotting at the protein level (**Figure 2B**), suggesting that this is a shared regulation across different types of cancers.

**Figure 3:**
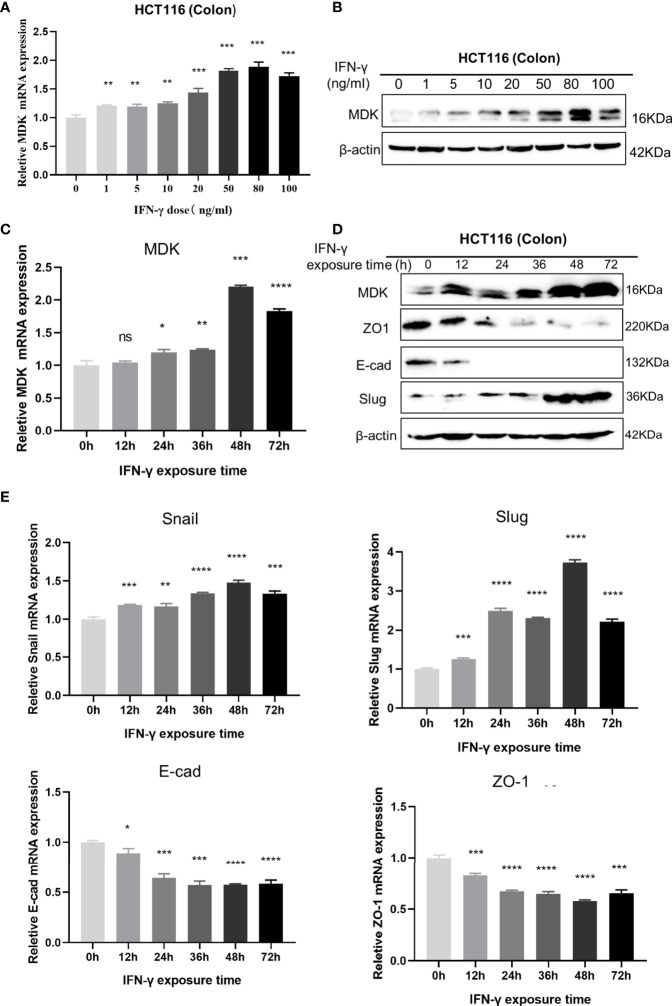
Dose- and time-dependent effects of IFN-γ on MDK induction. **(A)** RT-qPCR assays to examine the effect of IFN-γ concentration on MDK induction in the HCT116 cell line. **(B)** Western blotting assays to examine the effect of IFN-γ concentration on MDK induction in the HCT116 cell line. **(C)** RT-qPCR assays to examine the effect of IFN-γ treatment time on MDK induction in the HCT116 cell line. **(D)** Western blotting assays to examine the effect of IFN-γ treatment time on MDK induction and EMT activation in the HCT116 cell line. **(E)** RT-qPCR assays showing the effect of IFN-γ treatment time on EMT markers in the HCT116 cell line. *P < 0.05, **P < 0.01, ***P < 0.001, ****P < 0.0001. ns, not significant.

To further validate the IFN-γ-MDK regulatory axis, we treated the HCT116 cancer cell line with different concentrations of IFN-γ and verified that activation of MDK by IFN-γ was dose-dependent at both the mRNA (**Figure 3A**) and protein (**Figure 3B**) levels. Furthermore, IFN-γ treatment also caused an obviously time-dependent upregulation of MDK at both the mRNA (**Figure 3C**) and protein (**Figure 3D**) levels, as well as time-dependent activation of EMT evidenced by time-dependent downregulation of E-cad and ZO-1 but upregulation of Slug and Snail (**Figures 3D, E**).

### IFN-γ Activates MDK in a STAT1-Dependent Manner

**Figure 4:**
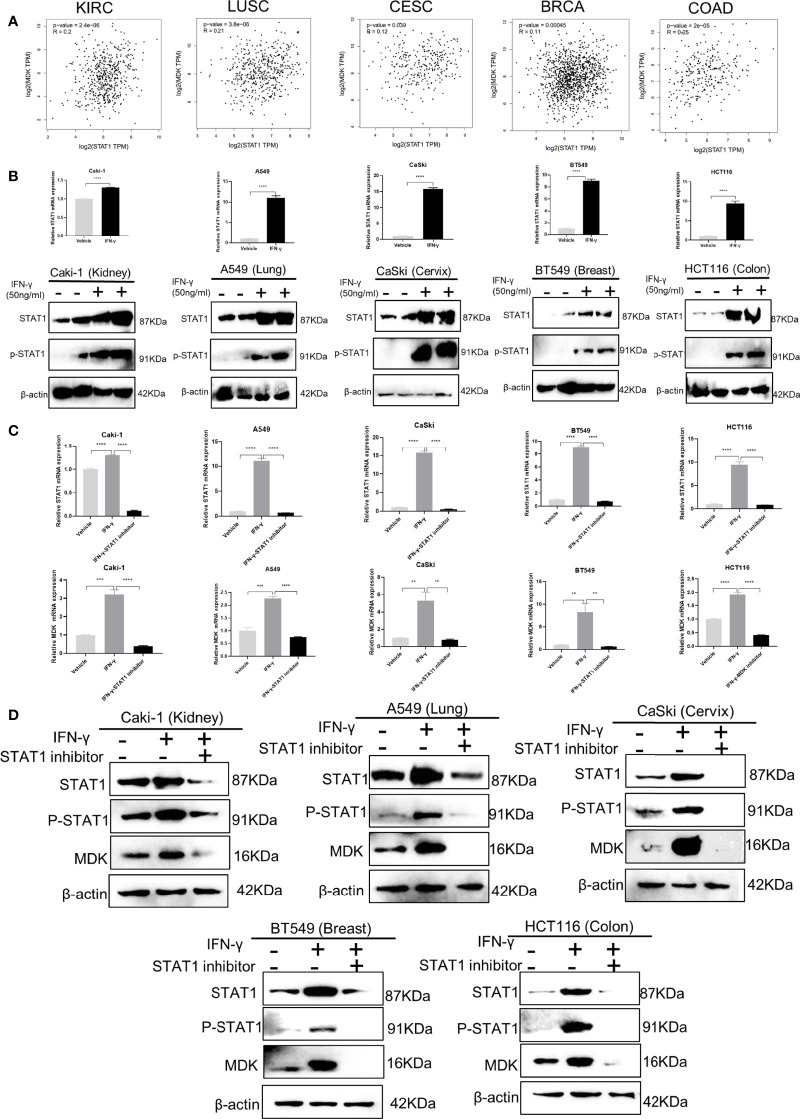
IFN-γ activates MDK *via* STAT1. **(A)** Correlation analyses using the GEPIA 2 online tool (http://gepia2.cancer-pku.cn/#correlation) to shown the correlation relationship between STAT1 and MDK using the RNA-seq data of different cancers from the Cancer Genome Atlas (TCGA) database. KIRC, kidney renal clear cell carcinoma; LUSC, Lung squamous cell carcinoma; CESC, Cervical squamous cell carcinoma and endocervical adenocarcinoma; BRCA, breast invasive carcinoma; COAD, colorectal adenocarcinoma. **(B)** RT-qPCR and Western blotting assays to detect the effect of IFN-γ treatment (50 ng/ml, 48h) on levels of STAT1 and phosphorylated STAT1 (p-STAT1) in five cancer cell lines. **(C)** RT-qPCR assays to the effect of STAT1 inhibitor on mRNA levels of STAT1 and MDK in five cancer cell lines. **(D)** Western blotting assays to examine the effect of STAT1 inhibitor on protein levels of STAT1, p-STAT1 and MDK in five cancer cell lines. Data are shown as the mean ± standard deviation (SD). **P < 0.01, ***P < 0.001, ****P < 0.0001.

STAT1 is a key downstream effector in the IFN-γ signalling (2), which leads us to speculate that the IFN-γ-MDK regulation may rely on IFN-γ-induced STAT1 activation. This speculation was preliminarily supported by the significant correlations between STAT1 and MDK in various cancers in the TCGA database (**Figure 4A**). Moreover, as reported, real-time qPCR and Western blotting assays showed that IFN-γ exposure caused a significant upregulation in levels of STAT1 abundance and phosphorylation (**Figure 4B**). Then we blocked STAT1 activation using a specific STAT1 inhibitor Fludarabine and assessed its influence on IFN-γ activation of MDK. Notably, STAT1 inhibitor remarkably diminished IFN-γ-induced STAT1 activation, and substantially abrogated IFN-γ-induced MDK activation at the mRNA level in all examined cell lines (**Figure 4C**), which was further validated at the protein level by Western blotting analyses (**Figure 4D**). All these demonstrate that IFN-γ activates MDK *via* STAT1 in cancer cells.

### MDK Promotes Cancer Metastasis by Activating the EMT Programme

**Figure 5:**
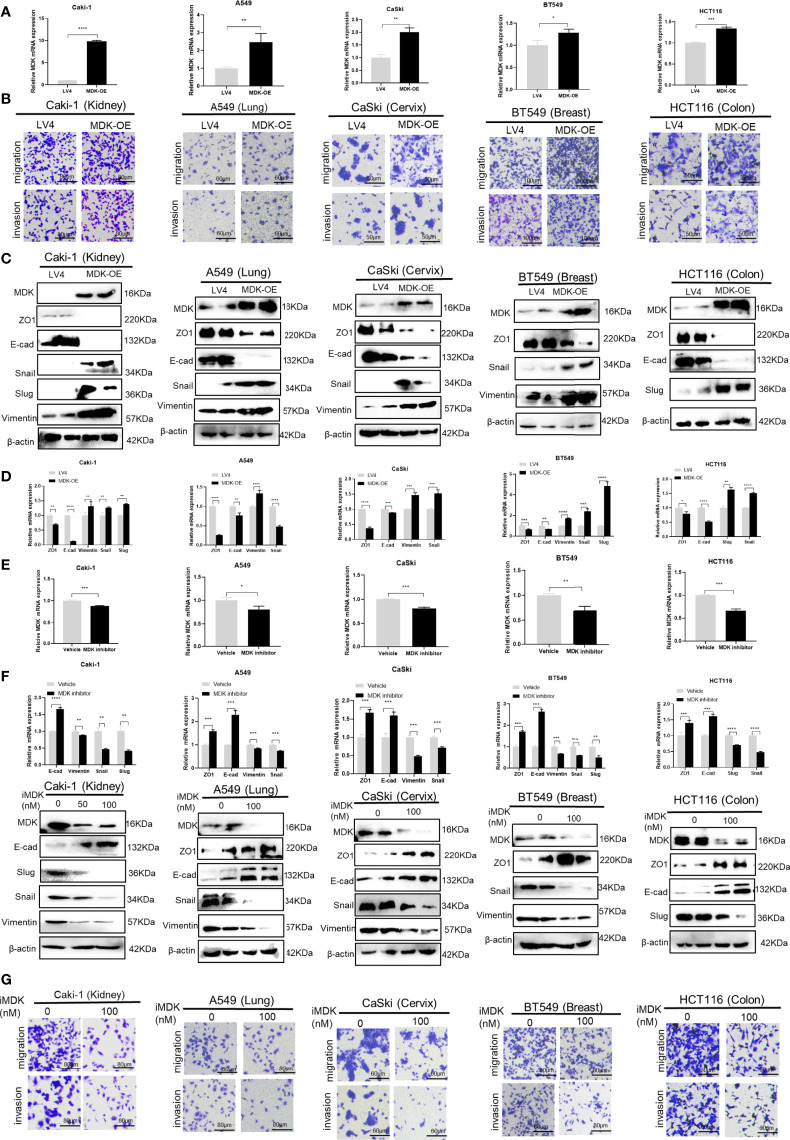
MDK promotes cancer metastasis by activating the EMT programme. **(A)** RT-qPCR validation of MDK overexpression in five cancer cell lines. **(B)** Transwell assays to assess the alteration of cell invasion and migration abilities by MDK overexpression in five cancer cell lines. **(C)** Western blotting assays to assess the effect of MDK overexpression on expression of EMT markers. **(D)** RT-qPCR assays to assess the effect of MDK overexpression on expression of EMT markers. **(E)** RT-qPCR validation of MDK inhibition by iMDK (100nM, 48h). **(F)** RT-qPCR and Western blotting assays to assess the effect of iMDK on EMT in five cancer cell lines. **(G)** Transwell assays to assess the effect of iMDK on cell invasion and migration in five cancer cell lines. Data are shown as the mean ± standard deviation (SD). *P < 0.05, **P < 0.01, ***P < 0.001, ****P < 0.0001.

To further explore the possibility that MDK is a shared mechanism underlying IFN-γ-triggered metastasis in various cancers, we first examined the effect of MDK on cancer metastasis in the above cancer cell lines. Transwell assays clearly showed that MDK overexpression (**Figure 5A**) enhanced the migration and invasion capacities of all five cancer cell lines (**Figure 5B**). In accordance, MDK overexpression resulted in activation of the EMT programme, as evidenced by loss of epithelial markers, and concomitant gain of mesenchymal markers at the both protein (**Figure 5C**) and mRNA (**Figure 5D**) levels. To further validate that MDK is necessary for cancer metastasis, we silenced MDK expression using a specific MDK inhibitor iMDK, which demonstrated high efficiency in decreasing endogenous MDK expression (**Figure 5E**) and subsequent EMT activation in all five cell lines (**Figure 5F**). In accordance, iMDK suppressed the migration and invasion in all five cell lines in transwell assays (**Figure 5G**). In line with its oncogenic role, MDK is elevated in the majority of cancer types in the TCGA dataset (**Figure S1**).

### Pharmacologically Targeting MDK Abrogates IFN-γ-Induced Metastasis

**Figure 6:**
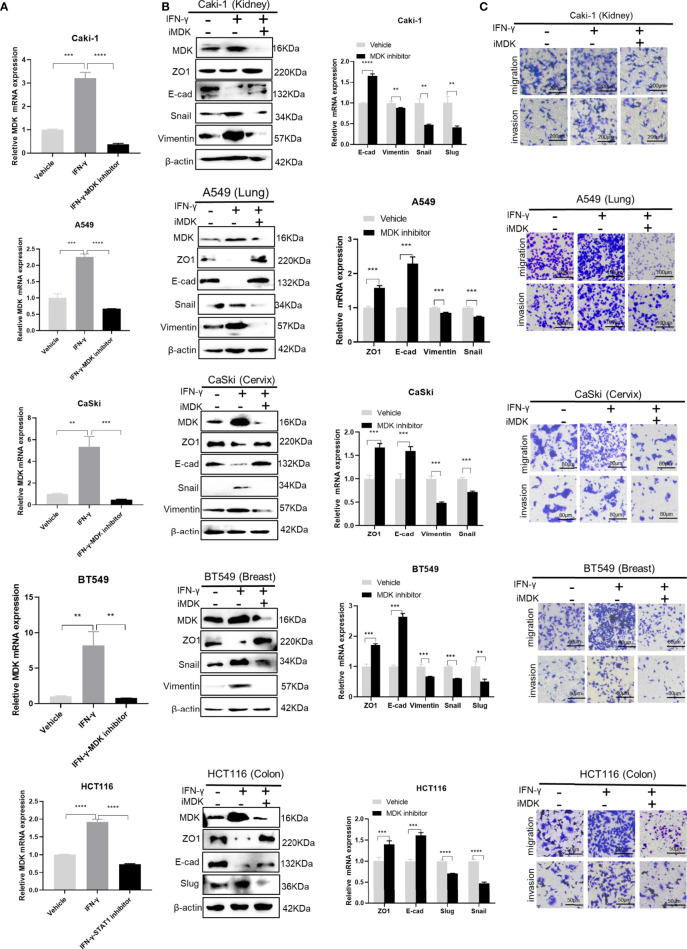
Targeting MDK abrogates IFN-γ-induced tumor metastasis. **(A)** RT-qPCR validation of MDK suppression by MDK inhibitor in IFN-γ-treated cancer cells. **(B)** Western blotting and RT-qPCR assays to assess the effect of iMDK on IFN-γ-activated EMT in five cancer cell lines. **(C)** Transwell assays to examine the effect of iMDK on IFN-γ-driven cell invasion and migration. Data are shown as the mean ± standard deviation (SD). **P < 0.01, ***P < 0.001, ****P < 0.0001.

To test whether MDK inhibition would attenuate IFN-γ-induced metastasis, we added iMDK (100 nM) to the IFN-γ-treated cancer cells, which resulted in no conspicuous cell death under microscope. As expected, iMDK efficiently eliminated IFN-γ-induced MDK expression at both the mRNA and protein levels (**Figures 6A, B**), reversed IFN-γ-driven EMT activation determined by Western blotting and RT-qPCR (**Figure 6B**), and subsequently abrogated IFN-γ-triggered migration and invasion as shown in transwell assays (**Figure 6C**) in all examined cancer cell lines. These data suggest that MDK confers the IFN-γ-elicited metastasis in various cancers, and that pharmacologically inhibiting MDK can broadly and efficiently abrogate IFN-γ treatment-induced cancer metastasis.

## Discussion

Accumulative evidence suggests that IFN-γ functions as a ‘‘two-edged sword’’ in cancer treatment (15, 23). IFN-γ is conventionally considered a promising cancer therapeutic agents for its potent tumoricidal and immunoregulatory activities, and certain positive responses have been reported in multiple pre-clinical and clinical trials (7). Tamura et al. reported that intralesional injection of IFN-γ could induce lasting remissions of T cell leukemia (8). Giannopoulos et al. demanstrated that intravesicle instillation of IFN-γ was effective in preventing bladder cancer recurrence (9). A randomized phase III trial showed that inclusion of IFN-γ in the first-line chemotherapy could prolong the progression-free survival of ovarian cancer patients (10).

Despite these encouraging results, a lack of beneficial effect has been observed in small-cell lung cancer, advanced colon cancer, metastatic renal cell carcinoma, and breast cancer (12, 15, 23). On the contrary, the pro-metastatic adverse effect of IFN-γ mediated by multiple mechanisms have been reported. Preincubation of murine colon adenocarcinoma cells with IFN-γ produced a significant increase in pulmonary metastases in mice (16). IFN-γ induced cancer invasiveness in prostate cancer *via* transcription of IFN-induced tetratricopeptide repeat 5 (IFIT5) (17). Low-dose IFN-γ enhanced the stemness and metastasis of non–small cell lung cancer *via* the intercellular adhesion molecule-1 (ICAM1)-PI3K-Akt-Notch1 axis (20). IFN-γ withdrawal after immunotherapy potentiates B16 melanoma invasion and metastasis by intensifying tumor integrin αvβ3 signaling (19). More recently, Singh et al. reported that loss of the tumor suppressive transcription factor Elf5 in triple-negative breast cancer mediated IFN-γ signalling-promoted tumor progression and metastasis (21).

Though the IFN-γ-mediated metastasis has been indicated in a variety of cancers, no shared mechanism has been revealed. Here, we verified that IFN-γ exerted its pro-metastatic effect *via* a common EMT activating mechanism in all examined cancer cell lines, including the kidney cancer Caki-1, the lung cancer A549, the cervical carcinoma CaSki, the breast cancer BT549, and the colon cancer HCT116. The EMT programme is a developmental program broadly promoting metastasis of various cancers. Activation of EMT enables cancer cells to lose their epithelial property but acquire a mesenchymal, migratory phenotype through downregulating epithelial markers including ZO-1 and E-cadherin and upregulating mesenchymal markers such as N-cadherin and Vimentin (24). In consistence with our finding, IFN-γ activation of EMT has also been reported in prostate cancer (17), suggesting that EMT activation is a common mechanism underlying IFN-γ-driven metastasis in different cancers.

To further deep into the mechanism mediating IFN-γ-triggered EMT activation, we focused on MDK, which is a heparin-binding growth factor and an emerging oncoprotein well implicated in induction of EMT and cancer metastasis (25). Indeed, we validated that MDK overexpression led to EMT and metastasis in all five cancer cell lines. MDK has been reported to promote cancer EMT *via* TGF-β, WNT and Notch 2 signalings (25). Additionally, MDK has also been implicated in promoting cancer proliferation (27), angiogenesis (28), stemness (29), drug resistance (30), immunosuppression (31), and resistance to immune checkpoint blockade (31), closely correlated with advanced cancer progression and poor survival (27, 32, 33).

Our data demonstrated that IFN-γ exposure resulted in a dramatic upregulation of MDK in all examined five cancer cell lines, suggesting this is a universal regulatory mechanism across cancers. To our knowledge, this is the first report demonstrating the common IFN-γ-MDK transduction cascade in the cancerous context. However, MDK was also previously reported to be elevated in peripheral blood lymphocytes in response to IFN-γ treatment when investigating its anti-HIV function (26), suggesting that this regulation may also exist in non-cancerous cells.

MDK is upregulated in the majority of cancers (at least 20 different cancer types), such as breast, lung, ovary, prostate, colon, gastric and pancreatic cancers, whereas its expression is generally low or undetectable in normal adult tissues (25, 34). However, the regulatory mechanism mediating MDK elevation is still largely obscure. There is evidence indicating that MDK is induced by hypoxia in a HIF-1α dependent manner (35). Here, we further indicate that MDK is also an IFN-γ inducible protein, which provides more insights into the MDK regulatory network in cancer.

STAT1 is the major IFN-γ-activated transcription factor responsible for the downstream signaling triggered by IFN-γ (2). The binding of IFN-γ to IFNGR1 triggers the formation of the IFNGR complex consisting of two IFNGR1 and two IFNGR2 subunits. This complex induces JAK1 and JAK2 phosphorylation, and further recruits and phosphorylates two STAT1 molecules. Phosphorylated STAT1 homodimerizes and translocates to the nucleus to initiate transcription of IFN-γ induced genes (3, 13). Our data demonstrated that STAT1 inhibition efficiently abrogated IFN-γ-induced MDK activation in all examined cancer cell lines, suggesting that the IFN-γ-MDK transduction is dependent on STAT1 in cancer.

To validate the role of MDK in mediating IFN-γ-activated EMT and metastasis, we pharmacologically silenced MDK expression using a specific inhibitor iMDK. The inhibitor iMDK is a small low molecular weight compound, demonstrating high efficiency and specificity in suppressing MDK expression in cancer cells (36, 37). iMDK decreased cell viability of the MDK-positive cancer cells in a dose-dependent manner, but less affected the MDK-negative cancer and non-transformed cells (36). The inhibitory effect of iMDK on cancer malignant behaviors has been validated in oral squamous cell carcinoma (37), non-small cell lung cancer (36, 38), and prostate cancer (39). Importantly, systemic administration of iMDK in mouse didn’t cause obvious systemic toxicity (36), highlighting a high safety for potential clinical use. Our data indicated that iMDK application could globally eliminate IFN-γ-induced MDK, reverse IFN-γ-induced EMT activation, and ultimately abrogate IFN-γ-triggered cancer migration and invasion in all examined cancer cell lines.

**Figure 7:**
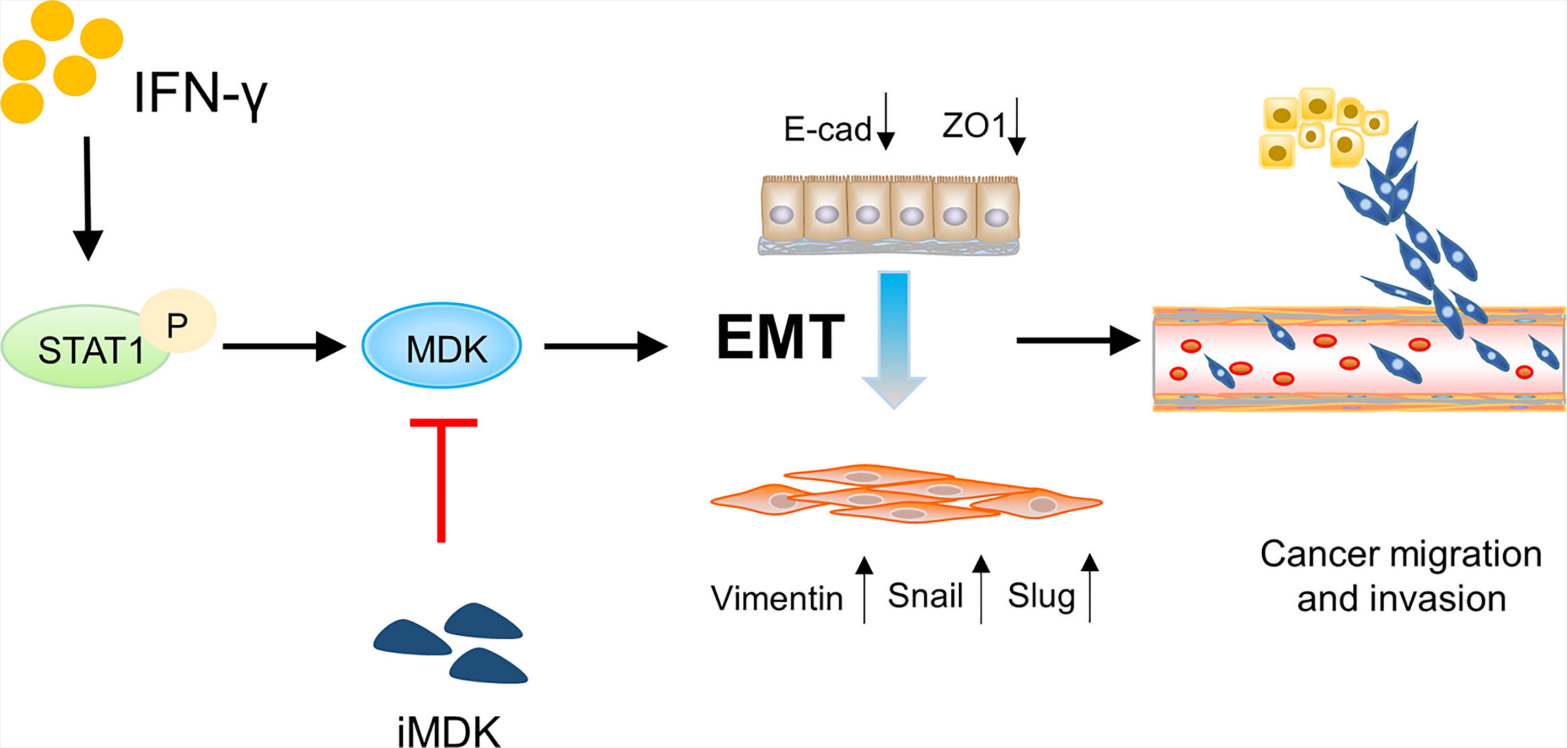
Schematic diagram showing the IFN-γ-STAT1-MDK signalling axis in driving cancer metastasis. The IFN-γ-STAT1-MDK signalling axis promotes cancer metastasis by activating EMT, whereas MDK inhibition by iMDK can abrogate IFN-γ-induced EMT and tumor metastasis.

Collectively, our data identify a novel IFN-γ-STAT1-MDK signalling axis (**Figure 7**), which confers the pro-metastatic adverse effect of IFN-γ in immunotherapy, whereas targeting MDK may efficiently abrogate IFN-γ-elicited cancer metastasis. Attenuation of the pro-metastatic activity of IFN-γ may help to augment its anti-tumor effects during cancer treatment, thus our data propose a combined use of MDK inhibitors in IFN-γ-based cancer therapies.

## Data Availability Statement

The original contributions presented in the study are included in the article/**Supplementary Material**. Further inquiries can be directed to the corresponding authors.

## Author Contributions

JL, TW, and CH designed and conceived the study. LZ, QL, RL, SC, JT, LL, and XD performed the experiments. LZ and QL analyzed the data. LZ and QL wrote the manuscript. JL and TW revised the manuscript. All authors contributed to the article and approved the submitted version.

## Funding

This work was financially supported by grants from the National Natural Science Foundation of China (No. 82172913, 82173234 and 82002754), and Open Funding of the State Key Laboratory of Molecular Oncology (SKLMO-KF2021-19).

## Conflict of Interest

The authors declare that the research was conducted in the absence of any commercial or financial relationships that could be construed as a potential conflict of interest.

## Publisher’s Note

All claims expressed in this article are solely those of the authors and do not necessarily represent those of their affiliated organizations, or those of the publisher, the editors and the reviewers. Any product that may be evaluated in this article, or claim that may be made by its manufacturer, is not guaranteed or endorsed by the publisher.

## Supplementary Material

Supplementary Figure 1 MDK is elevated in the majority of cancer types in the TCGA database. Red, cancer; blue, adjacent normal.
