# Supplementary figures and images for: Targeting MDK Abrogates IFN-γ-Elicited Metastasis inCancers of Various Origins
Source: Front Oncol. 2022 Jun 7;12:885656. doi: 10.3389/fonc.2022.885656 (PMC9210922; doi:10.3389/fonc.2022.885656)

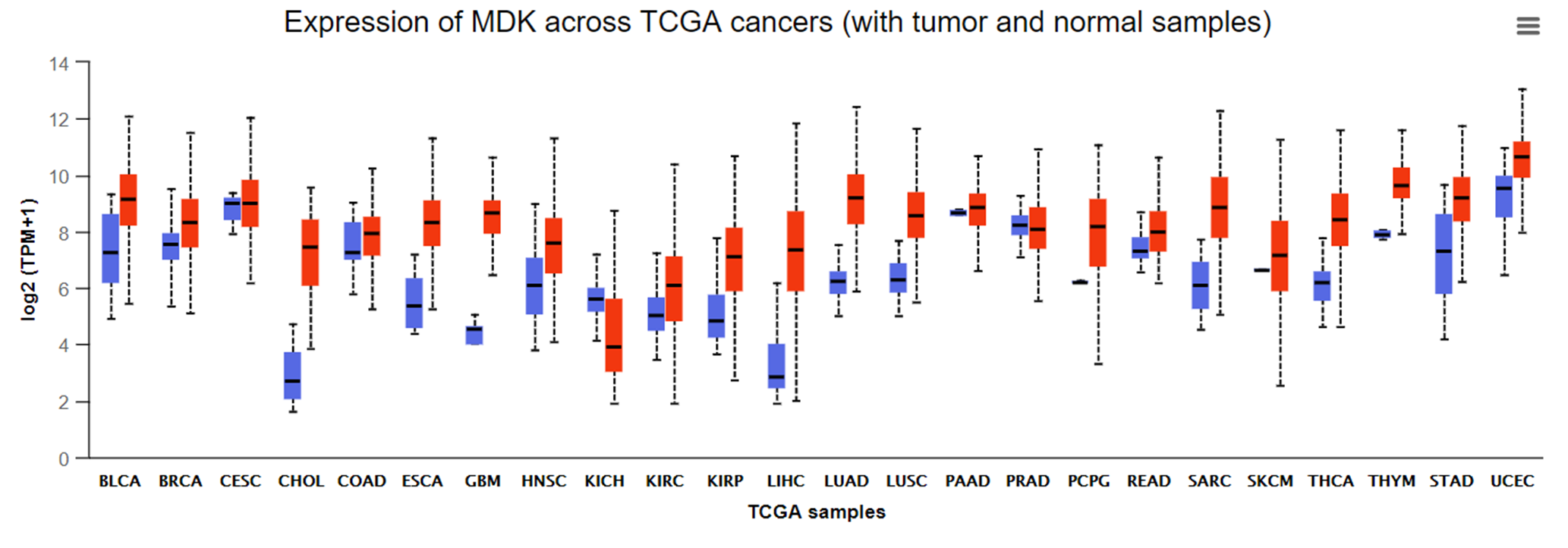

Supplement: Supplementary Figure 1 — MDK is elevated in the majority of cancer types in the TCGA database. Red, cancer; blue, adjacent normal. [file Image_1.tif]
