# Supplementary material for: Targeting MDK Abrogates IFN-γ-Elicited Metastasis inCancers of Various Origins
Source: Front Oncol. 2022 Jun 7;12:885656. doi: 10.3389/fonc.2022.885656 (PMC9210922; doi:10.3389/fonc.2022.885656)
Supplement: Supplementary file 2 [file Table_1.docx]

Table S1. Primer sequences

| Gene | Forward primer | Reverse primer |
| --- | --- | --- |
| MDK | 5’CGCGGTCGCCAAAAAGAAAG -3’ | 5’- TACTTGCAGTCGGCTCCAAAC -3’ |
| ZO1 | 5’- CAACATACAGTGACGCTTCACA -3’ | 5’- CACTATTGACGTTTCCCCACTC -3’ |
| E-cadherin | 5’- CGAGAGCTACACGTTCACGG -3’ | 5’- GGGTGTCGAGGGAAAAATAGG -3’ |
| Snail | 5’- AAGGCCTTCTCTAGGCCCT -3’ | 5’- CGCAGGTTGGAGCGGTCAG -3’ |
| Slug | 5’- CGAACTGGACACACATACAGTG -3’ | 5’- CTGAGGATCTCTGGTTGTGGT -3’ |
| Vimentin | 5’-AGTCCACTGAGTACCGGAGAC-3’ | 5’-CATTTCACGCATCTGGCGTTC-3’ |
| β-actin | 5’ -CATGTACGTTGCTATCCAGGC-3’ | 5’- CTCCTTAATGTCACGCACGAT -3’ |
| STAT1 | 5’-ATCAGGCTCAGTCGGGGAATA -3’ | 5’ -TGGTCTCGTGTTCTCTGTTCT -3’ |
